# Interseccionalidade e insegurança alimentar em favelas de Belo Horizonte, Minas Gerais, Brasil

**DOI:** 10.1590/0102-311XPT095724

**Published:** 2025-02-24

**Authors:** Karynna Maria da Silva Ferreira, Aline Dayrell Ferreira Sales, Uriel Moreira, Magda do Carmo Parajára, Amélia Augusta de Lima Friche, Waleska Teixeira Caiaffa, Elis Borde

**Affiliations:** 1 Faculdade de Medicina, Universidade Federal de Minas Gerais, Belo Horizonte, Brasil.

**Keywords:** Insegurança Alimentar, Interseccionalidade, Iniquidades em Saúde, Vulnerabilidade Social, Favelas, Food Insecurity, Intersectionality, Health Inequities, Social Vulnerability, Slums, Inseguridad Alimentaria, Interseccionalidad, Inequidades en Salud, Vulnerabilidad Social, Favelas

## Abstract

A insegurança alimentar gera graves implicações para a saúde e a vida das populações. Muitos determinantes sociais já foram identificados, mas a compreensão da insegurança alimentar ainda é limitada devido a uma visão fragmentada que desarticula as diferentes dimensões de vulnerabilidade. Este estudo objetiva analisar a (in)segurança alimentar sob o olhar da interseccionalidade, tendo como local de estudo duas favelas de Belo Horizonte (Minas Gerais, Brasil) e seus entornos. A análise transversal foi realizada com dados do inquérito domiciliar do Projeto BH-Viva. A variável de desfecho foi a insegurança alimentar, e as variáveis de exposição foram obtidas pela construção interseccional de indicadores de vulnerabilidade social (sexo, raça/cor e Índice de Vulnerabilidade Socioeconômica Sensível à Insegurança Alimentar - IVSIA, construído a partir dos domínios trabalho e renda, escolaridade e condições de domicílio). As associações entre a insegurança alimentar e as exposições foram estimadas por meio de modelos de regressão logística, e o efeito das categorias de interseccionalidade sobre a insegurança alimentar foi capturado por termos de interação apropriados. Entre as pessoas em condições socioeconômicas desfavoráveis (IVSIA), as mulheres negras apresentaram as maiores chances de apresentar insegurança alimentar (OR = 7,50; IC95%: 3,20-17,58) do que homens negros e mulheres brancas. Os resultados revelam que a insegurança alimentar é marcada por processos de vulnerabilização interseccionais, em que se sobrepõem os efeitos de privação socioeconômica, sexismo, patriarcado e racismo. Dessa forma, reforçam a necessidade de pesquisas com abordagens interseccionais para identificar os padrões e as principais vítimas da insegurança alimentar, bem como a urgência de políticas públicas voltadas para as necessidades desses grupos.

## Abstract (en)

Food insecurity has serious implications for populations’ health and lives. Many social determinants have been identified, but understanding food insecurity remains limited due to a fragmented view that segregates vulnerability dimensions. This study aims to analyze food (in)security based on an intersectional perspective, having two favelas in Belo Horizonte (Minas Gerais State, Brazil) and their surroundings as its studied site. This cross-sectional analysis was carried out with data from the household survey of the BH-Viva Project. Food insecurity was chosen as the outcome variable and the intersectional construction of social vulnerability indicators (sex, race/skin color, and the Socioeconomic Vulnerability Index Sensitive to Food Insecurity - IVSIA, constructed from the domains of work and income, education and housing conditions) was used as the exposure variables. The associations between food insecurity and the exposure variables were estimated by logistic regression models and the effect of the intersectionality categories on food insecurity was obtained by the appropriate interaction terms. Among people in unfavorable socioeconomic conditions (IVSIA), black women were more likely to have food insecurity (OR = 7.50; 95%CI: 3.20-17.58) than black men or white women. Results shows that food insecurity includes intersectional processes of vulnerability that overlap the effects of socioeconomic deprivation, sexism, patriarchy, and racism, thus reinforcing the need for intersectional research to find the patterns and main victims of food insecurity and the urgency of public policies toward these groups’ needs.

## Abstract (es)

La inseguridad alimentaria tiene graves implicaciones para la salud y la vida de las poblaciones. Ya se han identificado muchos determinantes sociales, pero la comprensión de la inseguridad alimentaria sigue siendo limitada debido a una visión fragmentada que desarticula las diferentes dimensiones de la vulnerabilidad. Este estudio tiene como objetivo analizar la (in)seguridad alimentaria desde la perspectiva de la interseccionalidad, teniendo como lugar de estudio dos favelas de Belo Horizonte (Minas Gerais, Brasil) y sus alrededores. El análisis transversal se realizó con datos de la encuesta domiciliaria del Proyecto BH-Viva. La variable de resultado fue la inseguridad alimentaria, y las variables de exposición se obtuvieron mediante la construcción interseccional de indicadores de vulnerabilidad social (género, raza/color e Índice de Vulnerabilidad Socioeconómica Sensible a la Inseguridad Alimentaria -IVSIA, construido a partir de los dominios de trabajo e ingresos, educación y condiciones de vivienda). Las asociaciones entre la inseguridad alimentaria y la exposición se estimaron mediante modelos de regresión logística, y el efecto de las categorías de interseccionalidad en la inseguridad alimentaria se capturó mediante términos de interacción apropiados. Entre las personas en situación socioeconómica desfavorable (IVSIA), las mujeres negras tenían más probabilidades de desarrollar inseguridad alimentaria (OR = 7,50; IC95%: 3,20-17,58) que los hombres negros y las mujeres blancas. Los resultados revelan que la inseguridad alimentaria estuvo marcada por procesos de vulnerabilidad interseccional, en los cuales se superponen los efectos de la privación socioeconómica, del sexismo, del patriarcado y del racismo. Por lo tanto, se refuerza la necesidad de investigación con enfoques interseccionales para identificar los patrones y las principales víctimas de la inseguridad alimentaria, así como la urgencia de políticas públicas dirigidas a las necesidades de estos grupos.

## Introdução

No Brasil, nos últimos anos (2016-2022), vivemos um período de desmonte de políticas sociais e de segurança alimentar devido a crises políticas e econômicas ^1^ ^,^ ^2^ ^,^ ^3^ ^,^ ^4^ ^,^ ^5^, com aumento expressivo da insegurança alimentar ^6^. Apesar dos avanços nas políticas entre 2004 e 2013, com benefícios significativos para a segurança alimentar ^2^ ^,^ ^3^ ^,^ ^7^ ^,^ ^8^, os grupos mais vulneráveis vivenciaram essas melhorias de maneira menos expressiva ^2^.

Dessa forma, a insegurança alimentar é profundamente marcada pelas iniquidades ^3^ ^,^ ^9^ ^,^ ^10^ historicamente estruturadas e reforçada pelo acesso desigual à saúde, à educação e a recursos ^1^ ^,^ ^3^ ^,^ ^8^, pela concentração de terras e exploração do trabalho ^3^, que levam a iniquidades persistentes no acesso a uma alimentação adequada e de qualidade ^8^.

Embora haja uma produção significativa sobre desigualdades sociais e insegurança alimentar no Brasil, ainda existem desafios em sua compreensão ^4^ ^,^ ^11^ ^,^ ^12^ ^,^ ^13^. As pesquisas da área ^2^ ^,^ ^5^ ^,^ ^12^ ^,^ ^13^ ^,^ ^14^ têm analisado a influência de distintos determinantes sociais, como escolaridade, renda, raça/cor, condições de moradia e sexo, na configuração e distribuição das desigualdades de insegurança alimentar. Entretanto, essas abordagens não têm permitido revelar o papel de processos sociais mais estruturais como racismo, sexismo e classismo, e o foco unidimensional e unitário ^15^ ^,^ ^16^ dificulta a compreensão das interações de diferentes processos de vulnerabilização que se sobrepõem para moldar a insegurança alimentar. Ainda que algumas pesquisas tenham avançado na compreensão dessa articulação e sua relação com a insegurança alimentar ^11^ ^,^ ^15^, incluíram apenas duas dimensões, como raça/cor e sexo.

Limitações semelhantes são associadas às perspectivas aditivas ^17^ ^,^ ^18^ ^,^ ^19^ que epistemologicamente reproduzem lógicas fatoriais e se restringem à adição dos efeitos e são interpretadas a partir de uma construção teórica incapaz de captar as dinâmicas de poder que definem vulnerabilidade e privilégio em territórios concretos ^20^.

Diante da complexidade social e política dos processos que (re)produzem/reforçam ^11^ ^,^ ^15^ ^,^ ^16^ ^,^ ^19^ a insegurança alimentar e limitam o acesso ao alimento, há reconhecimento da importância de desenvolver métodos mais avançados e, mais do que isso, mais sensíveis para avançar na compreensão da insegurança alimentar e seu enfrentamento.

Recentemente, a abordagem da interseccionalidade tem ganhado espaço nos estudos da saúde coletiva. Abrange a análise “*de como raça, gênero, deficiência, sexualidade, classe e outras categorias sociais são mutuamente moldadas e inter-relacionadas com forças históricas mais amplas, como colonialismo, neoliberalismo, geopolítica e configurações culturais para produzir mudanças nas relações de poder e opressão*” ^21^ (p. 1). Não é simplesmente um método de fazer pesquisa ^18^ ou uma técnica, mas envolve reorientar as perguntas de pesquisa ^22^ e adotar uma perspectiva metodológica e analítica capaz de proporcionar elementos para problematizar a distribuição desigual e reverter o quadro de iniquidades e injustiça. Piscitelli ^23^ (p. 268) afirma que “*raça, gênero e classe não são âmbitos diferentes de experiência que existem isoladamente uns dos outros, nem podem ser simplesmente montados em conjunto como se fosse um lego*”.

Diante disso, este estudo propõe analisar a prevalência e os padrões de insegurança alimentar em duas favelas ^24^ de Belo Horizonte (Minas Gerais) e em bairros de seus entornos a partir de uma abordagem interseccional. Mais especificamente, busca avançar na compreensão de como as múltiplas vulnerabilidades sociais se combinam para moldar a insegurança alimentar, utilizando, para isso, marcadores sociais para refletir processos mais amplos. Também se propõe explorar algumas implicações para o desenvolvimento metodológico e conceitual de estudos sobre insegurança alimentar na perspectiva da interseccionalidade para a formulação de políticas públicas sobre insegurança alimentar.

## Métodos

### Desenho e amostra do estudo

Estudo transversal com dados do Projeto BH-Viva ^25^, que buscou analisar os impactos das intervenções de transformação urbana implementadas pela Prefeitura Municipal de Belo Horizonte sobre a saúde e o bem-estar de moradores em zonas especiais de interesse social (ZEIS), aqui denominadas favelas ^24^. O BH-Viva é um projeto multifásico ^25^, sendo a fase II objeto deste estudo, composta por inquéritos domiciliares realizados no Aglomerado da Serra (2017), no Cabana do Pai Tomás (2018) e nos entornos de tais favelas ^25^. Uma amostragem por conglomerado em três estágios foi utilizada, sendo que: no primeiro estágio, foram sorteados os setores censitários; no segundo, domicílios particulares; e no terceiro estágio, foi sorteado um morador com mais de 18 anos para responder ao questionário. Adotou-se um nível de significância de 5%, estimativa de prevalência populacional de 35%, coeficiente de variação de 5% e 20% de perda, para o cálculo da amostra.

### Instrumento de coleta de dados

O instrumento de coleta de dados incluía questões, respondidas pelo morador maior de 18 anos, relacionadas ao domicílio (características de moradia, presença de menores de dez anos, renda e segurança alimentar) e características individuais (raça/cor, sexo, trabalho, escolaridade, benefícios sociais).

### Desfecho

A variável de desfecho foi a condição de insegurança alimentar a partir de quatro perguntas adaptadas da *Escala Brasileira de Insegurança Alimentar* (EBIA) ^26^ (Material Suplementar - Tabela S1; https://cadernos.ensp.fiocruz.br/static//arquivo/suppl-e00095724_7128.pdf), semelhante às versões reduzidas utilizadas em outros estudos ^27^ ^,^ ^28^ ^,^ ^29^.

A EBIA é usada pelo governo brasileiro para estimar as prevalências de insegurança alimentar desde 2003 ^3^ e mede experiências relacionadas à insegurança alimentar no domicílio ^30^. Para este estudo, foram consideradas as respostas fornecidas pelo adulto entrevistado, partindo do pressuposto de que ele seria capaz de informar a dinâmica familiar. De acordo com a escala, em segurança alimentar estariam aqueles com acesso regular e permanente a alimentos de qualidade e em quantidades suficientes; em insegurança alimentar leve, caso haja preocupação sobre acesso ao alimento em um futuro próximo e redução na qualidade da alimentação; em insegurança alimentar moderada aqueles com modificações nos padrões de alimentação aliadas à redução na quantidade de alimentos ingeridos, apenas entre os adultos; e insegurança alimentar grave quando houvesse comprometimento da qualidade e da redução da quantidade dos alimentos de todos os membros, podendo incluir a experiência de fome ^7^ ^,^ ^8^ ^,^ ^11^.

A condição relacionada à segurança alimentar foi classificada em duas categorias: (1) segurança alimentar/insegurança alimentar leve, para todos os entrevistados que responderam “não” a todas as perguntas ou apenas uma “sim”; e (2) insegurança alimentar moderada/grave, para todos os que responderam “sim” a duas ou mais perguntas. A junção das categorias foi realizada devido às semelhanças de construto entre os conceitos, a exemplo do que é feito em outros estudos da área ^13^ ^,^ ^14^ ^,^ ^15^.

### Variáveis de exposição

Os indicadores utilizados para refletir as dimensões e os eixos de marginalização (e privilégio) criados a partir da operacionalização de uma abordagem da interseccionalidade foram obtidos a partir do cruzamento entre gênero, raça/cor e vulnerabilidade socioeconômica (Material Suplementar - Figura S1; https://cadernos.ensp.fiocruz.br/static//arquivo/suppl-e00095724_7128.pdf).

Gênero foi aferido a partir da variável sexo: homem ou mulher. Embora tal classificação binária seja limitada para captar a diversidade de identidades e relações de gênero vinculadas às expressões do patriarcado e do sexismo, ela representa uma aproximação possível à dimensão de gênero no cenário de dados disponíveis.

Raça/cor foi aferida de acordo com as categorias do Instituto Brasileiro de Geografia e Estatística (IBGE), pela autodeclaração dos respondentes, diferenciando pretos, pardos, indígenas, amarelos e brancos.

Informada pela abordagem interseccional e pensando no contexto brasileiro, em que o racismo é marcado pelo colorismo, inicialmente criamos um modelo diferenciado, partindo da hipótese de encontrar diferenças entre pessoas autodeclaradas pretas e pardas. No entanto, em uma análise de sensibilidade, não detectamos diferença significativa entre essas pessoas quando comparadas com pessoas autodeclaradas brancas, no modelo sem interseccionalidade (M1A). Dessa forma, optamos por diferenciar brancos e negros (pessoas autodeclaradas como pretas e pardas). Optamos por excluir amarelos e indígenas das análises devido à baixa frequência de indivíduos nesses grupos. Ainda partimos da compreensão de que a inclusão desses dois grupos em uma categoria agregada de pessoas não brancas, por mais que seja útil para representar as dinâmicas do racismo em função da construção da branquitude no país, apagaria diferenças históricas entre tais categorias de raça/cor não brancas.

A vulnerabilidade socioeconômica foi mensurada a partir da construção de um Índice de Vulnerabilidade Socioeconômica Sensível à Insegurança Alimentar (IVSIA), especificamente criado para este estudo. Apesar de a renda ter um papel fundamental na mediação da insegurança alimentar ^1^ ^,^ ^8^ ^,^ ^15^, existem outros fatores ^1^ ^,^ ^2^ ^,^ ^7^ ^,^ ^8^ ^,^ ^10^ ^,^ ^12^ ^,^ ^13^ relacionados à classe social e às condições de domicílio que forjam as materialidades e experiências de vulnerabilidade socioeconômica e ultrapassam os limites conceituais e metodológicos da renda, especialmente em contextos de alta informalidade laboral. Em função da articulação interna entre as variáveis, ainda se observa que a consideração separada das dimensões de vulnerabilidade socioeconômica pode induzir a erros em sua interpretação ^31^. Assim, optamos pela construção do IVSIA, criado a partir da adaptação do índice de condições ambientais e moradia de Bittencourt et al. ^12^.

O IVSIA foi desenvolvido a partir de três domínios: trabalho e renda (três itens), escolaridade (um item) e condições de domicílio (nove itens) (Material Suplementar - Tabela S2; https://cadernos.ensp.fiocruz.br/static//arquivo/suppl-e00095724_7128.pdf). Uma pontuação foi atribuída para cada item, em que as situações favoráveis para a segurança alimentar receberam o valor 0 e as desfavoráveis receberam o valor 1 ^1^ ^,^ ^2^ ^,^ ^5^ ^,^ ^7^ ^,^ ^8^ ^,^ ^9^ ^,^ ^10^ ^,^ ^12^ ^,^ ^13^ ^,^ ^14^. O IVSIA foi, então, definido pela soma das pontuações de cada variável (valores entre 0 e 13) e classificado em duas categorias: “favorável para a segurança alimentar” quando seu valor era < 4 e “desfavorável para a segurança alimentar” quando seu valor era ≥ 4.

A partir do cruzamento dos indicadores, formaram-se oito categorias de interseccionalidade: “homem branco IVSIA favorável”, “homem negro IVSIA favorável”, “homem branco IVSIA desfavorável”, “homem negro IVSIA desfavorável”, “mulher branca IVSIA favorável”, “mulher negra IVSIA desfavorável”, “mulher branca IVSIA desfavorável”, “mulher negra IVSIA desfavorável”.

### Covariável

A variável de ajuste utilizada foi o Índice de Vulnerabilidade da Saúde (IVS) ^32^, indicador definido pela média ponderada de diferentes indicadores socioeconômicos (Material Suplementar; https://cadernos.ensp.fiocruz.br/static//arquivo/suppl-e00095724_7128.pdf) para cada setor censitário do município estudado, que aqui foi classificado como “baixo” (agrupamento das categorias “baixo risco” e “médio risco” do índice original) e “elevado” (agrupamento de “elevado risco” e “muito elevado”). Julgou-se necessário incluir o IVS para capturar as diferenças não observadas na insegurança alimentar entre as favelas e seus entornos em função do efeito socioeconômico contextual de cada setor censitário incluído no estudo.

### Análise estatística

Foi realizada a análise descritiva segundo características socioeconômicas e demográficas, e estimadas as prevalências de segurança alimentar/insegurança alimentar leve e da insegurança alimentar moderada/grave segundo as variáveis de exposição e categorias de interseccionalidade. As diferenças entre as prevalências foram avaliadas utilizando teste exato de Fisher.

Para aferir a validade da adaptação da versão curta do EBIA, foi verificado o critério de Kaiser-Meyer-Olkin (KMO), realizado o teste da esfericidade de Bartlett e conduzida análise fatorial exploratória (EFA; do inglês *exploratory factor analysis*). A consistência interna entre as quatro perguntas da escala foi medida pelo alfa de Cronbach. Para verificar a invariância da escala, conforme sugerido por Cezimbra et al. ^33^, foi realizada uma análise fatorial confirmatória (CFA; do inglês *confirmatory factor analysis*) para sexo, raça/cor e IVSIA. A análise de invariância foi conduzida utilizando a comparação entre modelos de equivalência configuracional, métrica e escalar por meio da qualidade do ajuste desses modelos, aferido pelo índice de ajuste comparativo (CFI; do inglês *comparative fit index*) e de testes do tipo razão de verossimilhanças, seguindo a abordagem de Bastos & Harnois ^34^. Valores de p maiores que 0,05 e reduções no CFI entre modelos não superiores a 0,002 foram tomados como indicação de invariância. Por conter quatro itens binários, a análise de validade da escala foi feita utilizando como base uma matriz de correlação policórica entre os itens ^35^.

Para aferir a adequação da categorização do IVSIA, foi realizada análise da associação simples do IVSIA com a insegurança alimentar utilizando o teste exato de Fisher. A validade do IVSIA foi aferida por meio do KMO, teste de Bartlett e condução de uma EFA, e sua consistência interna foi medida pelo alfa de Cronbach. Como os 13 itens do IVSIA são binários, a análise de validade e consistência também utilizou como base uma matriz de correlação policórica.

Posteriormente, foi estimada uma sequência de modelos logísticos com interação para aferir a associação entre a insegurança alimentar e as categorias de interseccionalidade. A sequência teve a seguinte forma: primeiro, três modelos com apenas uma das exposições (M1A - sexo, M1B - raça/cor, M1C - IVSIA); depois, três modelos com interações 2-a-2 entre as exposições (M2A - sexo e raça/cor, M2B - sexo e IVSIA, M2C - raça/cor e IVSIA); e finalmente, um modelo com todas as interações 2-a-2 e uma interação 3-a-3 (M3 - sexo, raça/cor e IVSIA). Todos os modelos foram ajustados pelo IVS do setor censitário correspondente. Os resultados dos modelos foram utilizados para calcular o efeito total e a prevalência correspondente a cada uma das categorias de interseccionalidade, e um teste global foi feito para verificar a significância conjunta dos termos de interação nos modelos M2A, M2B, M2C e M3. Após a estimação de cada modelo, foi analisado o fator de inflação de variância (VIF; do inglês *variance inflation factor*) para verificar o efeito da multicolinearidade sobre os resultados obtidos. Como análise de sensibilidade, o modelo M3 foi reestimado com a inclusão de um intercepto aleatório ao nível de setor censitário para aferir efeitos não-observados do contexto (setor censitário) sobre as estimativas.

As análises descritivas e de associação foram feitas com o software Stata, versão 17.0 (https://www.stata.com), as de validade e de invariância no ambiente R, versão 4.3 (http://www.r-project.org) com os pacotes *psych* e *lavaan*, respectivamente. O nível de significância adotado para todas as análises foi de 5%.

Questões éticas

A pesquisa foi aprovada pelo Comitê de Ética e Pesquisa da Universidade Federal de Minas Gerais (registro nº 11548913.3.0000.5149). Os entrevistados assinaram Termo de Consentimento Livre e Esclarecido (TCLE) antes de responder o questionário.

## Resultados

Para as análises descritivas, foram usados dados dos 1.194 participantes dos inquéritos domiciliares (72,1% favelas e 27,9% entorno). Mas, para as análises de associação, foram excluídas as respostas que apresentavam preenchimento incompleto das questões que compõem a EBIA (18%) e o IVSIA (38%). As perdas amostrais com relação à não-resposta não foram diferenciais, tanto para a EBIA quanto para o IVSIA (Material Suplementar; https://cadernos.ensp.fiocruz.br/static//arquivo/suppl-e00095724_7128.pdf).

Na análise de validade para a escala de insegurança alimentar construída neste estudo, os resultados foram adequados: o KMO foi de 0,82 (considerado ideal ^36^), o valor de p do teste de Bartlett foi < 0,001 (indicando que os dados são adequados para análise fatorial ^36^), as cargas fatoriais variaram de 0,85 a 0,97 na EFA com um fator, indicando uma excelente contribuição à escala ^37^; o percentual de variância explicada pelo primeiro fator na EFA com um fator foi de 85,5% e o alfa de Cronbach foi igual a 0,96, indicando alta consistência interna ^38^. Na análise de invariância da escala de insegurança alimentar por meio da CFA, foi observada um decréscimo menor que 0,002 no CFI entre os modelos de equivalência configuracional, métrica e escalar para os subgrupos de sexo, raça/cor e IVSIA. Os maiores valores de CFI foram observados no modelo de equivalência escalar, o que reforça a hipótese de invariância, indicando que a escala apresenta invariância escalar para sexo, raça/cor e IVSIA. Os valores de p dos testes χ^2^ para a diferença entre os modelos de equivalência foram maiores que 0,05 para todos os grupos, corroborando a invariância escalar (Material Suplementar - Tabela S3; https://cadernos.ensp.fiocruz.br/static//arquivo/suppl-e00095724_7128.pdf).

Na análise de validade para o IVSIA, o KMO foi de 0,53 (considerado medíocre ^36^), o valor de p do teste de Bartlett foi < 0.001 (indicando que os dados são adequados para análise fatorial ^36^) e das 13 variáveis que compõem o índice, quatro tiveram uma carga fatorial maior que 0,40 (renda *per capita*, material predominante do piso, rede de esgoto e superlotação) na EFA com um fator, o que indica que a contribuição das nove outras variáveis para o IVSIA são relativamente pequenas ^37^. Com relação à consistência do IVSIA, o alfa de Cronbach foi igual a 0,62, indicando baixa consistência interna ^38^. Embora os critérios de validade e consistência não tenham sido ideais, o IVSIA ainda assim apresentou associações significativas com insegurança alimentar, sejam elas não-ajustadas (Tabela 1) ou ajustadas (Tabelas 2, 3 e 4).

**Tabela 1:** Prevalência de segurança e insegurança alimentar, segundo características estudadas na população. Belo Horizonte, Minas Gerais, Brasil, 2017-2018.

Características	Segurança alimentar/insegurança leve		Insegurança alimentar moderada/grave		Valor de p *
	n	%	n	%	
Raça/Cor **					< 0,001
Brancos	178	76,1	56	23,9	
Negros	419	59,9	281	40,1	
IVSIA ***					< 0,001
Favorável	379	68,4	175	31,6	
Desfavorável	50	39,1	78	60,9	
Sexo					0,048
Feminino	346	61,7	215	38,3	
Masculino	244	68,2	114	31,8	
Menores de 10 anos residentes no domicílio					0,001
Sim	112	54,4	94	45,6	
Não	516	66,8	257	33,3	
IVS					0,020
Baixo ou médio risco	312	68,0	147	32,0	
Elevado ou muito elevado risco	316	60,8	204	39,2	

IVS: Índice de Vulnerabilidade da Saúde; IVSIA: Índice de Vulnerabilidade Socioeconômica Sensível à Insegurança Alimentar.

* Valor de p correspondente ao teste exato de Fisher;

** Foram excluídas as respostas de autodeclarados amarelos e indígenas (3% - 37 respostas);

*** O IVSIA foi construído a partir de três domínios: trabalho e renda, escolaridade e condições de domicílio.

**Tabela 2:** Associações entre insegurança alimentar e categorias de interseccionalidade. Belo Horizonte, Minas Gerais, Brasil, 2017-2018.

Características	M1A	M1B	M1C
OR (IC95%)	OR (IC95%)	OR (IC95%)
Sexo	1,39 (0,99-1,96)		
Raça/Cor	-	2,38 (1,56-3,64)	-
IVSIA	-	-	2,85 (1,86-4,38)
IVS	1,46 (1,05-2,05)	1,40 (0,99-1,97)	1,40 (0,99-1,98)
Efeitos totais (interações 2-a-2)			
Sexo#raça/cor	-	-	-
Sexo#IVSIA	-	-	-
Raça/cor#IVSIA	-	-	-
Efeitos totais (interações 3-a-3)			
Sexo#raça/cor#IVSIA	-	-	-

IC95%: intervalo de 95% de confiança; IVS: Índice de Vulnerabilidade da Saúde; IVSIA: Índice de Vulnerabilidade Socioeconômica Sensível à Insegurança Alimentar; OR: *odds ratio*.

Nota: os modelos são regressões logísticas com a insegurança alimentar como desfecho e sempre ajustados pelo IVS (referência: baixo ou médio risco). Todos os modelos utilizaram a mesma amostra, com informações completas para o desfecho, exposições e covariáveis. M1A: modelo apenas com sexo (referência: homens); M1B: modelo apenas com raça/cor (referência: brancos); M1C: modelo apenas com IVSIA (referência: IVSIA favorável).

**Tabela 3:** Associações entre insegurança alimentar e categorias de interseccionalidade. Belo Horizonte, Minas Gerais, Brasil, 2017-2018.

Características	M2A	M2B	M2C
OR (IC95%)	OR (IC95%)	OR (IC95%)
Sexo	1,23 (0,57-2,69)	1,28 (0,86-1,89)	-
Raça/Cor	2,17 (1,09-4,30)	-	2,14 (1,34-3,40)
IVSIA	-	2,14 (1,09-4,17)	2,37 (0,78-7,22)
IVS	1,39 (0,99-1,95)	1,39 (0,99-1,96)	1,34 (0,95-1,90)
Efeitos totais (interações 2-a-2)			
Sexo#raça/cor	3,11 (1,61-6,01)	-	-
Sexo#IVSIA	-	4,56 (2,50-8,32)	-
Raça/cor#IVSIA	-	-	5,73 (3,19-10,31)
Efeitos totais (interações 3-a-3)			
Sexo#raça/cor#IVSIA	-	-	-

IC95%: intervalo de 95% de confiança; IVS: Índice de Vulnerabilidade da Saúde; IVSIA: Índice de Vulnerabilidade Socioeconômica Sensível à Insegurança Alimentar; OR: *odds ratio*.

Nota: os modelos são regressões logísticas com a insegurança alimentar como desfecho e sempre ajustados pelo IVS (referência: baixo ou médio risco). Todos os modelos utilizaram a mesma amostra, com informações completas para o desfecho, exposições e covariáveis. M2A: modelo com sexo e raça/cor (referência: homens brancos), valor de p do teste global para interações = 0,0016; M2B: modelo com sexo e IVSIA (referência: homens com IVSIA favorável), valor de p do teste global para interações < 0,001; M2C: modelo com raça/cor e IVSIA (referência: brancos com IVSIA favorável), valor de p do teste global para interações < 0,001.

**Tabela 4:** Associações entre insegurança alimentar e categorias de interseccionalidade. Belo Horizonte, Minas Gerais, Brasil, 2017-2018.

Características	M3
OR (IC95%)
Sexo	0,99 (0,43-2,29)
Raça/Cor	1,76 (0,85-3,65)
IVSIA	0,73 (0,77-6,83)
IVS	1,33 (0,94-1,88)
Efeitos totais (interações 2-a-2)	
Sexo#raça/cor	2,40 (1,20-4,82)
Sexo#IVSIA	4,36 (1,01-18,88)
Raça/cor#IVSIA	3,94 (1,59-9,73)
Efeitos totais (interações 3-a-3)	
Sexo#raça/cor#IVSIA	7,50 (3,20-17,58)

IC95%: intervalo de 95% de confiança; IVS: Índice de Vulnerabilidade da Saúde; IVSIA: Índice de Vulnerabilidade Socioeconômica Sensível à Insegurança Alimentar; OR: *odds ratio*.

Nota: os modelos são regressões logísticas com a insegurança alimentar como desfecho e sempre ajustados pelo IVS (referência: baixo ou médio risco). Todos os modelos utilizaram a mesma amostra, com informações completas para o desfecho, exposições e covariáveis. M3: modelo com sexo, raça/cor e IVSIA (referência: homens brancos com IVSIA favorável).

Entre a população estudada, 35,9% viviam em insegurança alimentar moderada a grave e 19,3% estavam em posição desfavorável para o IVSIA. Com relação à raça/cor, 74,8% da população estudada se autodeclarava como preto ou pardo (26,2% e 48,5%, respectivamente). Aproximadamente 61,6% eram do sexo feminino e, em relação ao sexo e raça, observamos que a maioria era composta por mulheres negras (46,6%), seguida de homens negros (28,2%). Cerca de 22,2% com menores de dez anos no domicílio (Tabela 5).

**Tabela 5:** Caracterização demográfica e socioeconômica da população do estudo (respondentes). Belo Horizonte, Minas Gerais, Brasil, 2017-2018

Variáveis	n	%
Segurança alimentar		
Segurança alimentar/insegurança alimentar leve	628	64,2
Insegurança alimentar moderada/grave	351	35,9
Raça/Cor (autodeclarada) *		
Brancos	281	25,3
Negros (pretos e pardos)	832	74,8
IVSIA **		
Favorável	596	80,7
Desfavorável	143	19,3
Sexo		
Feminino	690	61,6
Masculino	431	38,5
Menores de 10 anos residentes no domicílio		
Sim	265	22,2
Não	929	77,8
IVS		
Baixo ou médio risco	548	45,9
Elevado ou muito elevado risco	646	54,1

IVS: Índice de Vulnerabilidade da Saúde; IVSIA: Índice de Vulnerabilidade Socioeconômica Sensível à Insegurança Alimentar.

* Foram excluídas as respostas de autodeclarados amarelos e indígenas (3% - 37 respostas);

** O IVSIA foi construído a partir de três domínios: trabalho e renda, escolaridade e condições de domicílio.

A prevalência de insegurança alimentar moderada/grave era maior entre pessoas negras comparadas com pessoas brancas (40,1% *vs.* 23,9%, p < 0,001), maior entre os em condições socioeconômicas desfavoráveis do que entre aqueles em condições mais favoráveis (60,9% *vs.* 31,6%, p < 0,001) e maior entre mulheres em comparação com homens (38,3% *vs.* 31,8%, p = 0,048). Observamos maior frequência de insegurança alimentar moderada/grave na presença de crianças menores de dez anos na residência (p < 0,05) (Tabela 1).

Com relação às regressões logísticas, nos modelos com apenas uma exposição, pessoas autodeclaradas negras e aqueles em condição desfavorável para o IVSIA apresentaram maior chance de insegurança alimentar moderada/grave do que brancos (OR = 2,38; IC95%: 1,56-3,64) e aqueles em condição favorável (OR = 2,85; IC95%: 1,86-4,38), respectivamente (Tabela 2). Nesses modelos, o VIF variou de 1,15 (M1C, IVSIA) a 1,76 (M1B, raça/cor), não indicando problemas devido à multicolinearidade ^39^.

Nos modelos com interações 2-a-2, homens negros (OR = 2,16; IC95%: 1,09-4,30) e mulheres negras (OR = 3,11; IC95%: 1,61-6,01) foram mais vulneráveis à insegurança alimentar moderada/grave que homens brancos. Homens em condições socioeconômicas desfavoráveis, segundo o IVSIA, têm chance duas vezes maior (OR = 2,14; IC95%: 1,09-4,17) e mulheres, nas mesmas condições, têm chance quatro vezes maior de sofrer insegurança alimentar (OR = 4,56; IC95%: 2,50-8,32) quando comparadas com homens em condições socioeconômicas favoráveis. Cabe ressaltar que pessoas negras em uma situação socioeconômica favorável ainda enfrentam maiores chances de insegurança alimentar do que pessoas brancas em condições semelhantes (OR = 2,14; IC95%: 1,34-3,40). Para pessoas negras, estar em uma condição socioeconômica desfavorável aumenta ainda mais o risco de insegurança alimentar (OR = 5,73; IC95%: 3,19-10,31) (Tabela 3). Nesses modelos, o VIF variou de 1,63 (M2B, IVS) a 7,69 (M2C, interação entre raça/cor e privação), não indicando problemas devido à multicolinearidade ^39^.

No modelo com interação 3-a-3, quando comparadas com homens brancos em situação socioeconômica favorável, mulheres negras (OR = 2,40; IC95%: 1,20-4,82), nas mesmas condições, mulheres brancas (OR = 4,36; IC95%: 1,01-18,88) e homens negros (OR = 3,94; IC95%: 1,59-9,73) em condições desfavoráveis tiveram maiores chances de insegurança alimentar. A categoria de interseccionalidade para a qual encontramos associação mais forte com a insegurança alimentar foi a das mulheres negras em condições socioeconômicas desfavoráveis, as quais têm uma chance sete vezes maior de vivenciar insegurança alimentar em comparação com os homens brancos em condições socioeconômicas favoráveis (OR = 7,50; IC95%: 3,20-17,58) (Tabela 4). Nesse modelo, o VIF variou de 2,01 (IVS) a 19,42 (interação entre sexo, raça/cor e IVSIA). Apesar de valores maiores que 10 para o VIF usualmente representarem um motivo de alerta, nos modelos estimados o efeito da multicolinearidade não foi suficiente para afetar a significância das principais associações ^39^.

O modelo M3 nos permite ainda estimar a prevalência de insegurança alimentar moderada/grave de cada uma das categorias de interseccionalidade. Centralizando os ajustes em suas respectivas médias, temos: homens brancos com IVSIA favorável (p = 21,6%), mulheres brancas com IVSIA favorável (p = 21,5%), homens negros com IVSIA favorável (p = 32,6%), homens brancos com IVSIA desfavorável (p = 16,7%), mulheres negras com IVSIA favorável (p = 39,8%), mulheres brancas com IVSIA desfavorável (p = 54,6%), homens negros com IVSIA desfavorável (p = 52,1%) e mulheres negras com IVSIA desfavorável (p = 67,4%).

Na análise de sensibilidade, separando os efeitos da raça/cor sobre a insegurança alimentar entre pretos e pardos (quando comparados aos brancos), ambos tiveram uma maior chance de enfrentar insegurança alimentar (pretos: OR = 2,82; IC95%: 1,71-4,65 pretos; pardos: OR = 1,94; IC95%: 1,24-3,06); porém, a diferença entre esses (com relação aos brancos) não foi significativa (OR = 1,45; IC95%: 0,97-2,17). Na análise de sensibilidade feita para aferir efeitos contextuais não-observados, não foram detectadas diferenças qualitativas e nem quantitativas entre o modelo M3 e o modelo multinível correspondente (em particular, a variância estimada do intercepto aleatório ao nível de setor censitário foi igual a 2,25×10^-33^).

## Discussão

Este estudo focou na análise das relações interseccionais dos eixos raça/cor, gênero e vulnerabilidade socioeconômica na insegurança alimentar. Verificou-se alta prevalência de insegurança alimentar moderada/grave, comparada com a registrada por pesquisas recentes em escala nacional e estadual ^6^ ^,^ ^40^, e evidenciou marcadas iniquidades em diferentes posições interseccionais, as quais revelam processos específicos de vulnerabilização, mas também de privilégio relativo. Considerando o contexto do município estudado, pioneiro na execução de políticas públicas em prol da garantia de segurança alimentar e nutricional ^41^ ^,^ ^42^, como o fornecimento de refeições, promoção da agricultura familiar e urbana e educação alimentar e nutricional ^43^, os resultados reforçam a necessidade de um olhar mais atento às desigualdades intraurbanas e ao alcance territorial e social das políticas desenvolvidas no município.

Nosso estudo revelou que pessoas negras apresentaram maior chance de insegurança alimentar quando comparadas com pessoas brancas. Essa tendência se observa entre mulheres e homens e, também, entre aqueles em condições socioeconômicas favoráveis. Esses resultados estão alinhados com os achados de pesquisa anterior ^7^, a partir de dados da *Pesquisa Nacional por Amostra de Domicílios*, que indicou que chefes de domicílios que se autodeclararam não brancos foram mais propensos à insegurança alimentar moderada/grave, e dados da *Pesquisa de Orçamentos Familiares*, que demonstrou que, em todas as regiões do país, a proporção de domicílios com pessoa de referência autodeclarada negra, a insegurança alimentar moderada/grave foi maior do que em domicílios nos quais a pessoa de referência se autodeclarou branca ^15^. Chaparro et al. ^44^ identificam o racismo estrutural como um dos principais impulsionadores da insegurança alimentar entre as famílias negras no Brasil. Dado que o racismo “*estrutura profundamente a nossa sociedade*” ^45^ (p. 1), integra sua organização econômica e política e “*se manifesta por meio de práticas conscientes ou inconscientes que culminam em desvantagens*” ^46^ (p. 26) para a população negra. Essas famílias enfrentam, assim, barreiras históricas que, por sua vez, irão determinar “*o local de moradia e a possibilidade de acessar ou não direitos à justiça, a bens e a serviços de saúde*” ^45^ (p. 1), além de enfrentar maior dificuldade de acesso ao mercado de trabalho e a oportunidades ^45^ ^,^ ^46^.

Não observamos associação significativa ao comparamos mulheres com homens, o que possivelmente se relaciona à centralidade da questão racial na influência da insegurança alimentar nos locais de estudo, bem como às limitações referentes à variável disponível no inquérito (sexo e não gênero). Contudo, também é possível que homens, ao não serem majoritariamente responsáveis pelo preparo da comida em função dos tradicionais papéis de gênero, podem não estar tão cientes da condição de (in)segurança de sua residência.

Em condições de alta vulnerabilidade socioeconômica, as mulheres, quando comparadas aos homens, tiveram maior chance de insegurança alimentar moderada/grave, como também evidenciado por estudos nacionais e internacionais ^7^ ^,^ ^15^ ^,^ ^47^. Esse fato destaca um paradoxo amplamente reconhecido: mulheres frequentemente enfrentam maior risco de insegurança alimentar, apesar de desempenharem um papel crucial na promoção da segurança alimentar nas comunidades e famílias. As desigualdades de gênero se manifestam em diversos âmbitos, incluindo o acesso ao mercado de trabalho ^7^, em desigualdades salariais ^7^ ^,^ ^47^ e na desigual divisão do trabalho, mais especificamente, do trabalho doméstico e do cuidado com implicações para a insegurança alimentar ^14^. Estudos apontam que a probabilidade de insegurança alimentar moderada/grave aumenta em domicílios com três ou mais crianças, naqueles com morador menor de 18 anos ^9^ ^,^ ^13^ e menor de cinco anos ^10^. Em nosso estudo, observamos a prevalência de insegurança alimentar moderada/grave era maior nos domicílios com crianças. Essa realidade pode estar relacionada a maiores despesas devido às suas necessidades específicas, bem como ao fato de que as mulheres frequentemente assumem o trabalho de cuidado, o que pode limitar sua capacidade de assumir trabalhos remunerados.

A literatura nacional ^1^ ^,^ ^7^ ^,^ ^9^ ^,^ ^10^ ^,^ ^14^ aponta que fatores como menor posse de bens de consumo; baixa escolaridade materna; baixa renda; desemprego; recebimento de benefícios sociais; e condições inadequadas do domicílio estão fortemente ligados à insegurança alimentar. Essas questões vão impactar a “*perpetuação de um ciclo intergeracional de poucas oportunidades, o que limita o desenvolvimento do capital humano nesses espaços*” ^7^ (p. 11), descrito pelos níveis de nutrição, saúde, educação e de investimentos nessas áreas ^7^ ^,^ ^48^. Nos resultados deste estudo, isso se manifesta, por exemplo, ao encontrarmos que situações desfavoráveis relacionadas à renda *per capita*, recebimento de benefícios sociais e superlotação domiciliar foram associadas a maiores percentuais de insegurança alimentar moderada/grave.

As desigualdades no acesso à alimentação refletem o fato evidenciado neste e em demais estudos ^11^ ^,^ ^13^ ^,^ ^15^ em que mulheres negras se apresentam mais vulneráveis à insegurança alimentar. As materialidades e experiências de vulnerabilidade que marcam as vidas das mulheres negras “periféricas” das favelas analisadas se resumem na insegurança alimentar que persiste apesar dos esforços políticos direcionados à garantia da segurança alimentar e nutricional no município ^41^ ^,^ ^42^. Dessa forma, os resultados desafiam leituras homogeneizadoras e simplistas das favelas como lugares de insegurança alimentar e traçam um perfil diferenciado das principais vítimas da insegurança alimentar que se articulam nas intersecções entre as relações de gênero, do racismo e das vulnerabilidades socioeconômicas. Especificamente, envolvem processos vinculados à feminização da pobreza, ao racismo estrutural e institucional, assim como à divisão desigual do trabalho.

A leitura interseccional permitiu identificar processos protetores vinculados aos privilégios historicamente consolidados mesmo em contextos de relativa desvantagem nas favelas e seus entornos, analisados neste estudo. Assim, detectamos uma maior chance de insegurança alimentar em mulheres em condições socioeconômicas desfavoráveis e mulheres em condições favoráveis, quando comparadas aos homens. Contudo, mulheres brancas em condições socioeconômicas desfavoráveis apresentaram uma chance menor de insegurança alimentar do que as mulheres negras nas mesmas condições, refletindo privilégios vinculados à branquitude que podem mediar, por exemplo, o acesso a recursos. A semelhança evidencia articulações interseccionais complexas nas quais se sobrepõem privilégios e vulnerabilidades e se configuram experiências marcadas pela minimização ou até mesmo a neutralização de benefícios em função do racismo, e se evidencia que a pobreza ou precariedade econômica não são equalizadores para todos os grupos sociais. De forma semelhante, Silva et al. ^11^ (p. 9) apontaram que a “*mulher branca vivencia as discriminações de gênero. Mas sua característica racial possibilita maiores oportunidades dentro das relações sociais*”, e argumentam que a “*diversificação de funções sociais não foi estendida à mulher negra, mas reforçou desigualdades de classe e raça entre as mulheres, pois as atividades que não foram culturalmente trabalhadas dentro do ambiente doméstico foram transferidas para mulheres negras, reforçando a presença desse grupo em ambiente privado, na posição, por exemplo, de empregadas domésticas*” ^11^ (p. 9).

Os resultados deste estudo apontam que ser mulher, negra e estar em condição socioeconômica desfavorável aumentou expressivamente a chance de insegurança alimentar moderada/grave. Convergindo com Dias et al., que apresentaram questões importantes sobre a insegurança alimentar a partir de uma análise do diário de Carolina Maria de Jesus (2014), trata-se de uma “*espécie de imbricação entre o ser mulher, o ser preta e o ser pobre*” ^49^ (p. 6) e da “*permanência colonial mais cruel*” ^49^ (p. 9) que não se restringe ao acesso reduzido a recursos econômicos necessários para adquirir alimentos, mas envolve processos mais estruturantes da consolidação histórica do país. Destaca-se, nesse sentido, a concentração da terra, o êxodo rural e as implicações do modelo agroexportador para a insegurança alimentar, assim como os padrões de urbanização que formaram as grandes cidades brasileiras e marcam a polarização social urbana.

Este estudo se propôs a trazer uma leitura interseccional que partiu da articulação entre gênero, raça/cor e vulnerabilidade socioeconômica e buscou explorar como as múltiplas vulnerabilidades sociais se combinam para moldar a insegurança alimentar no Aglomerado da Serra e no Cabana do Pai Tomás e seus entornos. Buscamos, dessa forma, superar algumas limitações metodológicas e conceituais que problematizamos no início do artigo e propor uma leitura que permite ampliar o olhar sobre as relações de poder e suas expressões na configuração da insegurança alimentar no município.

Entretanto, é importante reconhecer algumas limitações deste estudo. Primeiro, cabe dizer, com relação ao desfecho, que a utilização de uma adaptação da EBIA e a classificação em categorias agregadas (segurança alimentar/insegurança alimentar leve e insegurança alimentar moderada/ grave) podem ter apagado importantes dimensões da insegurança alimentar, mesmo que tenha sido testada quanto à validade, consistência e invariância. E, adicionalmente é razoável sopesar um possível aumento na magnitude do desfecho estudado considerando a pandemia da COVID-19, ocorrida após a coleta dos dados deste estudo. Segundo, optamos por utilizar dados dos respondentes e não dos chefes da família para a análise, já que para eles não havia dados para construção do IVSIA e nem de cor de pele. Terceiro, cabe mencionar que a operacionalização da abordagem da interseccionalidade para um estudo que inicialmente não foi pensado a partir de uma pergunta interseccional gerou desafios de ordem conceitual e metodológica. Dessa forma, não foi possível captar importantes elementos, como a orientação sexual e a identidade de gênero ou detalhes sobre os trabalhos de cuidado de dependentes e deficiências. Por fim, reconhecemos as limitações para a generalização e comparação dos resultados, tanto pelo uso da EBIA reduzida quanto pela característica da amostra estudada.

## Conclusão

Com relação às pesquisas, os resultados do estudo destacam o potencial que representa uma análise interseccional para compreender os padrões da insegurança alimentar e entender como não só as múltiplas vulnerabilidades sociais, mas também os privilégios, se combinam para moldar a insegurança alimentar e a segurança alimentar, respectivamente. A análise proposta neste estudo buscou ampliar o alcance da análise interseccional para além do cruzamento de duas variáveis (como sexo e raça) ao partir de um modelo ampliado de oito categorias interseccionais. Além dos desafios metodológicos que foram apontados anteriormente, cabe concluir que uma análise interseccional da insegurança alimentar e de qualquer outro desfecho em saúde deve necessariamente passar por uma teorização da interseccionalidade devido à coexistência de diversas abordagens que utilizam os mesmos termos, mas podem ter implicações metodológicas e analíticas diferentes.

Para políticas públicas, os resultados do estudo destacam a necessidade de esforços específicos para garantir a segurança alimentar de mulheres negras em condições socioeconômicas desfavoráveis, por elas e provavelmente também seus filhos serem as principais vítimas da insegurança alimentar. Educação infantil com vagas para o período integral podem, por exemplo, ser estratégicas para promover a segurança alimentar das mulheres negras socioeconomicamente vulnerabilizadas e seus dependentes. Outras estratégias podem envolver a assistência em saúde e a detecção precoce de insegurança alimentar, focalizando especificamente mulheres negras.

Estudos que tragam esse olhar ampliado e interseccional são extremamente necessários para o direcionamento de políticas públicas, permitindo, assim, construir ações efetivas na busca da equidade, da justiça social, da redução das desigualdades e da insegurança alimentar em nosso país.
