# Supplementary material for: Intersectionality and food insecurity in favelas in Belo Horizonte, Minas Gerais State, Brazil
Source: Cad Saude Publica. 2025 Feb 24;41(1):e00095724. [Article in Portuguese] doi: 10.1590/0102-311XPT095724 (PMC11863631; doi:10.1590/0102-311XPT095724)
Supplement: Supplementary file 1 [file 1678-4464-csp-41-01-PT095724-s.pdf]

# MATERIAL SUPLEMENTAR

## EBIA

A variável de desfecho foi a condição de IA a partir de quatro perguntas adaptadas da Escala Brasileira de Insegurança Alimentar e Nutricional (EBIA) contidas no questionário do Projeto BH-Viva (Tabela S1):

**Tabela S1** Perguntas adaptadas da EBIA contidas no questionário do Projeto BH-Viva

| Número da pergunta | Pergunta |
|---|---|
| **1** | Os moradores deste domicílio tiveram a preocupação de que os alimentos acabassem antes de poderem comprar ou receber mais comida? |
| **2** | Os alimentos acabaram antes que os moradores deste domicílio tivessem dinheiro para comprar mais comida? |
| **3** | Os moradores deste domicílio ficaram sem dinheiro para ter uma alimentação saudável e variada? |
| **4** | Os moradores deste domicílio comeram apenas alguns alimentos que ainda tinham por que o dinheiro acabou? |

## IVSIA

O Índice de Vulnerabilidade Socioeconômica sensível à Insegurança Alimentar (IVSIA) foi criado a partir de três domínios. O domínio trabalho e renda foi composto pelas variáveis: (a) renda per capita (desfavorável caso fosse menor que 1/2 do salário-mínimo, e favorável caso contrário); (b) recebimento de benefícios sociais (desfavorável caso recebesse algum benefício e favorável caso contrário); e (c) trabalha ou não trabalha (desfavorável caso estivesse desempregado, caso contrário favorável). Para o domínio escolaridade utilizamos: (d) escolaridade da mãe (desfavorável caso essa tenha cursado apenas até a 4ª série e favorável caso contrário). Para o domínio condições do domicílio: (e) material predominante na parede: favorável se alvenaria ou alvenaria sem revestimento e desfavorável caso contrário; (f) material predominante da cobertura (favorável se telha ou laje e desfavorável caso contrário); (g) material predominante do piso (desfavorável se cimento, madeira aproveitada ou terra e favorável caso contrário); (h) fornecimento de água (favorável se rede geral de distribuição e desfavorável caso contrário); (i) domicílio próprio (favorável se próprio e desfavorável caso contrário); (j) superlotação ou densidade morador/cômodo (desfavorável se $\geq 3$ e favorável caso contrário); (l) esgoto (favorável se rede coletora de esgoto ou pluvial e desfavorável caso contrário); (m) coleta de lixo (favorável se coletado diretamente por serviço de limpeza público por caminhão e desfavorável caso contrário); (n) geladeira (desfavorável se nenhuma e favorável caso contrário). "A definição do ponto de corte do IVSIA em menor que 4 (favorável para a SA) ou maior ou igual a 4 (desfavorável para a SA) foi baseada na literatura científica e foram analisadas a sua associação com a insegurança alimentar". A caracterização da população conforme dados utilizados para construção do IVSIA encontra-se na Tabela S2.

**Tabela S2** Caracterização da população conforme dados utilizados para construção do Índice de Vulnerabilidade Social sensível a Insegurança Alimentar (IVSIA). Belo Horizonte, Minas Gerais, Brasil, 2017-2018.

| **Variáveis** | **n** | **%** |
|---|---|---|
| **Trabalho e renda** | | |
| Renda per capita | | |
| Favorável | 532 | 61,4 |
| Desfavorável | 334 | 38,6 |
| Recebimento de benefícios sociais | | |
| Favorável | 1.012 | 86,4 |
| Desfavorável | 160 | 13,6 |
| Trabalha | | |
| Favorável | 537 | 46,0 |
| Desfavorável | 630 | 54,0 |
| **Escolaridade** | | |
| Escolaridade da mãe | | |
| Favorável | 260 | 25,4 |
| Desfavorável | 765 | 74,6 |
| **Condições do domicílio** | | |
| Material predominante na parede do domicílio | | |
| Favorável | 1.042 | 89,2 |
| Desfavorável | 126 | 10,8 |
| Material predominante na cobertura do domicílio | | |
| Favorável | 1.148 | 97,5 |
| Desfavorável | 29 | 2,5 |
| Material predominante no piso do domicílio | | |
| Favorável | 1.081 | 91,8 |
| Desfavorável | 96 | 8,2 |
| Fornecimento de água | | |
| Favorável | 1.162 | 98,9 |
| Desfavorável | 13 | 1,1 |
| Domicílio próprio | | |
| Favorável | 994 | 84,4 |
| Desfavorável | 184 | 15,6 |
| Pessoas no domicílio por cômodo (superlotação) | | |
| Favorável | 926 | 98,6 |
| Desfavorável | 13 | 1,4 |
| Rede de esgoto | | |
| Favorável | 1.156 | 98,5 |
| Desfavorável | 18 | 1,5 |
| Coleta de lixo | | |
| Favorável | 1.010 | 85,8 |
| Desfavorável | 167 | 14,2 |
| Possui geladeira | | |
| Favorável | 1.146 | 97,9 |
| Desfavorável | 25 | 2,1 |

## Perdas amostrais

As perdas amostrais com relação à não-resposta não foram diferenciais, tanto para a EBIA quanto para o IVSIA. Com relação ao sexo, as proporções de homens e mulheres na amostra completa eram de 38,45% e 61,55%, respectivamente, e essas proporções foram muito similares para os não-respondentes da EBIA (36,14% e 63,86%, respectivamente) e do IVSIA (38,45% e 61,55%, respectivamente). Com relação à raça-cor, as proporções de brancos e negros na amostra completa eram de 25,25% e 74,75%, respectivamente, e essas proporções também foram muito similares para

os não-respondentes da EBIA (26,26% e 73,74%, respectivamente) e do IVSIA (23,17% e 76,83%, respectivamente).

**IVS**

O Índice de Vulnerabilidade da Saúde (IVS) do município de Belo Horizonte foi utilizado para ajuste dos modelos finais. O IVS é composto por duas dimensões e 8 variáveis: Saneamento (percentual de domicílios particulares permanentes com abastecimento de água inadequado ou ausente; percentual de domicílios particulares permanentes com esgotamento sanitário inadequado ou ausente; e percentual de domicílios particulares permanentes com destino do lixo de forma inadequada ou ausente) e Socioeconômica (razão de moradores por domicílio; percentual de pessoas analfabetas; percentual de domicílios particulares com rendimento per capita até ½ SM; rendimento nominal mensal médio das pessoas responsáveis (invertido); percentual de pessoas de raça/cor preta, parda e indígena).

**Operacionalização da interseccionalidade**

Para melhor representar a operacionalização da interseccionalidade para este estudo apresentamos abaixo uma representação gráfica da articulação dos indicadores gênero, raça/cor e vulnerabilidade socioeconômica e outras questões relacionadas que não puderam ser contempladas pelo mesmo.

**Figura S1** Representação gráfica da articulação de múltiplas desigualdades relevantes para pensar o contexto brasileiro e mais especificamente, para o contexto do município de Belo Horizonte na perspectiva interseccional.

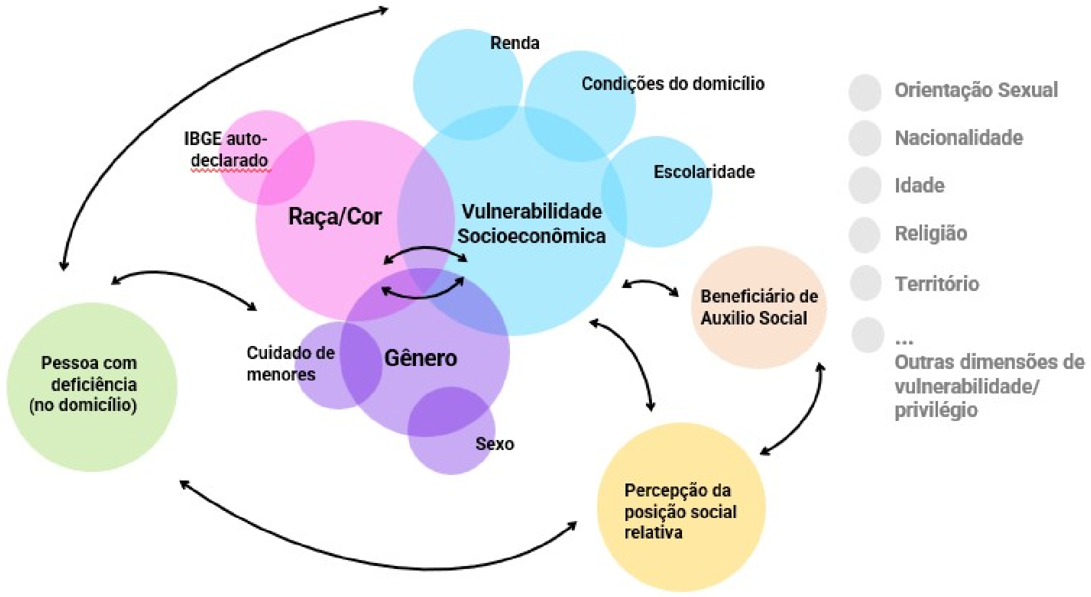

**Análise de invariância da EBIA adaptada**

A Tabela S3 abaixo contém os resultados detalhados da análise de invariância da escala de IA, construída a partir das quatro perguntas retiradas da EBIA apresentadas no questionário do Projeto BH-Viva.

**Tabela S3** Análise da invariância entre os subgrupos de sexo, raça/cor e IVSIA. Belo Horizonte, Minas Gerais, Brasil, 2017-2018.

| **Equivalência de mensuração** | **Métricas da qualidade de ajuste dos modelos** | | | | **Comparação entre modelos (valor de p)** | |
|---|---|---|---|---|---|---|
| | **χ2** | **gl** | **p** | **CFI** | **Métrica vs. configuracional** | **Escalar vs. métrica** |
| Sexo | | | | | | |
| Configuracional | 21,388 | 2 | < 0,001 | 0,998 | - | - |
| Métrica | 16,530 | 5 | 0,005 | 0,999 | 0,390 | - |
| Escalar | 11,862 | 6 | 0,06 | 0,999 | - | 0,962 |
| Raça/Cor | | | | | | |
| Configuracional | 23,434 | 2 | < 0,001 | 0,997 | - | - |
| Métrica | 16,348 | 5 | 0,006 | 0,999 | 0,330 | - |
| Escalar | 11,762 | 6 | 0,067 | 0,999 | - | 0,967 |
| IVSIA | | | | | | |
| Configuracional | 19,966 | 2 | < 0,001 | 0,998 | - | - |
| Métrica | 16,108 | 5 | 0,007 | 0,999 | 0,329 | - |
| Escalar | 12,034 | 6 | 0,061 | 0,999 | - | 0,559 |

Para a análise de invariância, foi utilizada a amostra completa, isto é, sem observações faltantes para as variáveis de IA, sexo, raça-cor e IVSIA (n = 612).

O p-valor das comparações entre os modelos de invariância métrica vs. configuracional e escalar vs. métrica corresponde ao teste da razão de verossimilhanças realizado entre esses modelos.

χ2 = estatística qui-quadrado (dividida pelos graus de liberdade), gl = graus de liberdade correspodnentes e p = p-valor do teste da razão de verossimilhanças para a diferença entre o modelo correspondente e um modelo saturado; CFI = "Índice de Ajuste Comparativo" (do inglês, Comparative Fit Index).
